# Modeling a linkage between blood transcriptional expression and activity in brain regions to infer the phenotype of schizophrenia patients

**DOI:** 10.1038/s41537-017-0027-3

**Published:** 2017-09-07

**Authors:** El Chérif Ibrahim, Vincent Guillemot, Magali Comte, Arthur Tenenhaus, Xavier Yves Zendjidjian, Aida Cancel, Raoul Belzeaux, Florence Sauvanaud, Olivier Blin, Vincent Frouin, Eric Fakra

**Affiliations:** 1 Aix-Marseille Univ, CNRS, CRN2M Marseille, France; 2 Fondation FondaMental, Fondation de Recherche et de Soins en Santé Mentale, Créteil, France; 3 Aix-Marseille Univ, CNRS, INT, Inst Neurosci Timone, Marseille, France; 4 INSERM, U 1127 Paris, France; 5 CNRS, 7225 Paris, France; 6 Sorbonne Universités, UPMC Univ Paris 06, UMRS_1127 Paris, France; 7 ICM, Département des maladies du système nerveux and Département de Génétique, Hôpital Pitié-Salpêtrière, Paris, France; 8 Laboratoire des Signaux et Systèmes (L2S, UMR CNRS 8506), CentraleSupélec-CNRS Université Paris-Sud, Gif-sur-Yvette, France; 9 Bioinformatics/Biostatistics Platform IHU-A-ICM, Brain and Spine Institute, Paris, France; 10 Pôle Psychiatrie centre, Hôpital de la Conception, Assistance Publique des Hôpitaux de Marseille, Marseille, France; 11 Service Hospitalo-Universitaire de Psychiatrie Secteur Saint-Etienne, Hôpital Nord, Saint-Etienne, France; 12 McGill Group for Suicide Studies, Douglas Mental Health University Institute, Department of Psychiatry, McGill University, Montreal, Quebec, Canada; 13 CIC-UPCET et Pharmacologie Clinique, Hôpital de la Timone, Assistance Publique des Hôpitaux de Marseille, Marseille, France; 14 CEA, DSV/I2BM, NeuroSpin, Gif-sur-Yvette, France

## Abstract

Hundreds of genetic loci participate to schizophrenia liability. It is also known that impaired cerebral connectivity is directly related to the cognitive and affective disturbances in schizophrenia. How genetic susceptibility and brain neural networks interact to specify a pathological phenotype in schizophrenia remains elusive. Imaging genetics, highlighting brain variations, has proven effective to establish links between vulnerability loci and associated clinical traits. As previous imaging genetics works in schizophrenia have essentially focused on structural DNA variants, these findings could be blurred by epigenetic mechanisms taking place during gene expression. We explored the meaningful links between genetic data from peripheral blood tissues on one hand, and regional brain reactivity to emotion task assayed by blood oxygen level-dependent functional magnetic resonance imaging on the other hand, in schizophrenia patients and matched healthy volunteers. We applied Sparse Generalized Canonical Correlation Analysis to identify joint signals between two blocks of variables: (i) the transcriptional expression of 33 candidate genes, and (ii) the blood oxygen level-dependent activity in 16 region of interest. Results suggested that peripheral transcriptional expression is related to brain imaging variations through a sequential pathway, ending with the schizophrenia phenotype. Generalization of such an approach to larger data sets should thus help in outlining the pathways involved in psychiatric illnesses such as schizophrenia.

## Imaging: Searching for links to aid diagnosis

Researchers explore links between the expression of genes associated with schizophrenia in blood cells and variations in brain activity during emotion processing. El Chérif Ibrahim and Eric Fakra at Aix-Marseille Université, France, and colleagues have developed a method to relate the expression levels of 33 schizophrenia susceptibility genes in blood cells and functional magnetic resonance imaging (fMRI) data obtained as individuals carry out a task that triggers emotional responses. Although they found no significant differences in the expression of genes between the 26 patients with schizophrenia and 26 healthy controls they examined, variations in activity in the superior temporal gyrus were strongly linked to schizophrenia-associated gene expression and presence of disease. Similar analyses of larger data sets will shed further light on the relationship between peripheral molecular changes and disease-related behaviors and ultimately, aid the diagnosis of neuropsychiatric disease.

## Introduction

Schizophrenia (SCZ) is a severe psychiatric disorder arising from complex and dynamic interactions between genetic and environmental factors. SCZ has a strong genetic component with heritability estimated up to 80% based on family and twin studies.^1^ In the recent years, concomitant advances in genomic technologies and massive increase in sample sizes (tens of thousands of DNA samples) through large consortia allowed mega genome-wide association study (GWAS) analysis that identified over 100 common variants conveying risk for SCZ at conventionally accepted standards of significance.^2^ However, how these genetic risk variants influence brain activity to lead to SCZ phenotype remains elusive.

An alternative strategy to uncover the neurophysiological impact of risk genes is the search of endophenotypes. Endophenotypes, or intermediate phenotypes, are defined as quantifiable stable biological variations or deficits that represent trait markers or indicators of vulnerability to a disease.^3^ The underlying assumption is that susceptibility genes for SCZ do not directly encode for clinical syndrome, but rather affect basic physiological process, as the development of neural systems for instance, that instigate the emergence of disease clinical symptoms.^4^ Among critical features of SCZ, emotional disturbances impose significant consequences on clinical trajectory and functional outcome. Indeed, the aberrant emotional responses observed in SCZ may result from impaired activity in the cortico-limbic regions that support emotional processing.^5^

Imaging genetics has proved to be effective in highlighting brain imaging variations that form the links between disease-related clinical and behavior traits and the associated vulnerability genes.^6^ Initially, the genetic information associated to imaging recordings in SCZ was focused on DNA sequence variations whose functional impact was assessed from post mortem brain tissue RNA.^7, 8^ Nevertheless, recent investigations demonstrate the meaningful links between genetic data obtained from readily assayed peripheral tissues and regional brain reactivity examined using blood oxygen level-dependent (BOLD) functional magnetic resonance imaging (fMRI).^9–12^

It has been hypothesized that gene expression was the most fundamental step where the genotype may critically impact the SCZ phenotype. In this line, altered mRNA profiling has been conducted in brain tissues as well as from peripheral blood from SCZ patients compared to controls.^13–16^ Studying mRNAs, the final step of gene transcription, allows bypassing all the regulatory processes (e.g. epigenetic and co-transcriptional mechanisms) that influence gene expression. Several reports indicated that peripheral blood cells, even though they constitute an indirect measure of the central nervous system, shared significant similarities with tissues from multiple brain regions on a transcriptional expression level. Such cells could thus serve as valuable probe to assess brain metabolism.^17^ The overlap between neural and peripheral blood cells might be a consequence of a common epigenetic dysregulation operative inducing similar patterns of DNA methylation across tissues.^18^ Indeed, direct comparison of gene expression profiles within brain tissue and peripheral blood cells of SCZ patients revealed numerous classes of genes, including so-called SCZ susceptibility genes that were common to both tissues and allowed for the identification of shared biological pathways.^19, 20^ In addition, even if abnormalities in blood were not a perfect mirror of pathological processes in brain, they could also represent distinct molecular changes that are specific to the primary pathophysiology or even reflect responses that are secondary to the disease.^21^ Therefore, whether or not mimicked in brain, transcriptional alterations in blood could be potential indicators of disease pathology.^22^

The unprecedented surge in data acquisition in biomedical imaging and genomics’ field represent computational and statistical challenges to identify the few genetic loci underlying phenotypic variations in brain function and structure. Indeed, two blocks of heterogeneous data need to be computed: (i) the pattern of gene expression and (ii), the neuroimaging phenotypes. To correlate these data, a common strategy is to run univariate regressions between all possible voxels and fold changes (FC) and adjust for multiple comparisons. Such an approach presents the advantage being straightforward. Though, it doesn’t take into account the correlation structure among FC and voxels. In addition, the incremental number of tests performed would undoubtedly lack power. Accordingly, different methods had to be developed to identify joint signals in a pair of high-dimensional data sets and to distinguish the few variables in the first block, which correlated with a few variables from a second block.^23, 24^ Recently, we developed such a method, Sparse Generalized Canonical Correlation Analysis (SGCCA) as a generalization of the Canonical Correlation Analysis. SGCCA allows to jointly examine a set of more than two blocks of heterogeneous data, while taking into account a structural design describing the relationships between these blocks.^25^ SGCCA has been successfully applied to several kinds of multi-block data sets.^25–27^

In this context of translational emerging explorations of the intricate links between peripheral molecular changes, brain function and behavioral responses, we proposed to use SGCCA to explore how variations in the transcriptional expression of candidate genes and BOLD activity in specific regions of interest (ROI) might infer the complex phenotype of SCZ patients. The 33 candidate genes tested in the present study were selected on the basis of a previous meta-analysis we conducted, that pointed out differential expressions between healthy controls and SCZ patients.^19^ Also, these genes were involved in the biological processes most frequently associated with SCZ such as immune/inflammatory function and the major histocompatibility complex region (*ADGRE1, CXCR3*, *CX3CR1*, *FYN*, *HLA-A*, *HLA-C*, *IFITM3*, *IL1B*, *IL2RB*, *NFKBIA*, *PRF1*, *S100A8*, *SRGN*), brain development and neurotransmission (*EOMES*, *ADGRG1*, *PPT1*, *S100A10*, *SLC6A4*, *TCF4*), metabolism (*ABL1*, *FTO*, *GYG1*, *TCN1*, *UBE2D2*), cell cycle/death (*G3BP2*, *MBD4*, *MT1X*, *MT2A*, *MTMR6*, *RAB6A*), MAPK cascade (*ELK1*, *MAPK6*), and regulation of transcription (*CEBPD*, *DR1*).^28–32^ Some of these candidate genes (*CEBPD*, *ELK1*, *FTO*, *FYN*, *IL1B*, *NFKBIA*, *S100A10*, *SLC6A4*, *TCF4*) have also been implicated in modulation of emotion, or/and fear, or/and attention or/and memory,^33–43^ processes stimulated during the fMRI task we applied. A few genes have also been genetically associated to SCZ and response to psychotropic drugs (*HLA-A*, *HLA-C*, *SLC6A4*, *TCF4*).^2, 44–48^ Finally, in line with the dominating neurodevelopmental hypothesis for SCZ, we explored the temporal dynamics of transcription in human prefrontal cortex through the braincloud application^49^ and the BrainSpan atlas for whole brain.^50^ About half of the gene candidates we selected exhibited either transcriptional decrease of expression in brain (*ABL1*, *DR1*, *EOMES*, *FYN*, *TCF4*) or transcriptional increase during fetal and first years of development (*GYG1*, *HLA-A*, *HLA-C*, *IFITM3*, *MT1X*, *MT2A*, *PPT1*, *S100A10*, *SRGN*). Regarding brain function, we used a previously validated fMRI emotional paradigm, the Variable Attention and congruency Task [VAAT], specifically designed to elicit the cortico-limbic system involved in emotion processing, as well as perceptual areas recruited in visual processing.^51^

## Results

### Differential analysis

We first examined how blood transcriptional expression of 33 candidate genes as well as the BOLD activity in the 16 ROI could distinguish the SCZ group (*N* = 29) from the HC group (*N* = 33). Statistical linear models taking into account the effect of age, sex and smoking covariates were carried out (Table 1). Whereas no candidate gene achieved significance (only a trend was observed with *S100A10* and *CX3CR1*), bilateral dorsolateral prefrontal cortex (DLPFC), superior temporal gyrus (STG), anterior cingulate cortex (ACC) as well as the left fusiform gyrus (FG) differed significantly between SCZ and healthy control groups. To assess the effect of treatment on the RNA expression and the BOLD signal, we ran a differential analysis in the patients only, and no significant effect was observed (data not shown). To complement gene-by-gene and ROI-by-ROI univariate comparisons in the differential analysis we also performed multivariate (e.g. MANCOVA) analyses, allowing us to analyze the overall difference between patients and controls in the RNA and imaging blocks of data while controlling for age, gender and the smoking status. When we examined the functional imaging block of data, we observed a significant effect of the factor of interest (e.g. Group, *Pr( > F)* = 0.015) whereas there is no effect for age, gender and smoking (Supplementary Table 1). This suggests that there is a direct link between the imaging data and the psychiatric phenotype of the tested subjects. By contrast, the MANCOVA applied to the block of RNA candidate gene expression did not reveal any significant effect for factor of interest (*Pr( > F)* = 0.308) as well as for the covariates (Supplementary Table 2). Thus, this result supports the fact that peripheral RNA candidate gene expression is not directly related to the phenotype of the studied individuals. Because the total number of variables from both blocks of imaging and RNA expression data exceeds the limit of validity of the MANCOVA procedure, we could not conclude for any synergistic effect of RNA and imaging with such method. Therefore, we need to explore alternative methods that are technically adapted to a situation where the number of variables exceeds the number of individuals and more importantly are able to conclude as to whether there is a combined correlation between RNA and imaging data to explain the difference between patients and controls.

**Table 1:** Capacity of candidate genes expression and ROIs intra-connectivity to separate SCZ and healthy subjects

Variable	raw P-value	adjusted P-value
*right DLPFC*	7.28E-5	3.79E-3
*right STG*	2.02E-4	5.26E-3
*dorsal ACC*	8.29E-4	1.44E-2
*left DLPFC*	4.76E-3	4.93E-2
*rostral ACC*	5.06E-3	4.93E-2
*left FG*	5.68E-3	4.93E-2
*left STG*	7.63E-3	5.67E-2
*S100A10*, S100 calcium binding protein A10	6.33E-2	4.12E-1
*CX3CR1*, Chemokine (C-X3-C motif) receptor 1	9.09E-2	5.25E-1
*MAPK6*, Mitogen-activated protein kinase 6	1.66E-1	7.88E-1
*right amygdala*	1.80E-1	7.88E-1
*right PG*	1.97E-1	7.88E-1
*RAB6A*, RAB6A Member RAS oncogene family	2.43E-1	9.02E-1
*left amygdala*	2.74E-1	9.13E-1
*G3BP2*, GTPase activating protein (SH3 domain) binding protein 2	2.92E-1	9.13E-1
*MT1X*, Metallothionein 1X	3.58E-1	9.13E-1
*SRGN*, Serglycin	4.31E-1	9.13E-1
*TCF4*, Transcription factor 4	4.32E-1	9.13E-1
*right FG*	4.44E-1	9.13E-1
*ELK1*, ELK1 member of ETS oncogene family	4.59E-1	9.13E-1
*IL1B*, Interleukin 1 beta	4.65E-1	9.13E-1
*left thalamus*	4.67E-1	9.13E-1
*CXCR3*, Chemokine (C-X-C motif) receptor 3	4.71E-1	9.13E-1
*FTO*, Fat mass and obesity associated	5.08E-1	9.13E-1
*right thalamus*	5.13E-1	9.13E-1
*PRF1*, Perforin 1	5.53E-1	9.13E-1
*HLA-A*, Major Histocompatibility Complex Class I A	5.54E-1	9.13E-1
*IFITM3*, Interferon induced transmembrane protein 3	5.54E-1	9.13E-1
*UBE2D2*, Ubiquitin-conjugating enzyme E2 D2	5.92E-1	9.13E-1
*right IOG*	5.97E-1	9.13E-1
*EOMES*, Eomesodermin	6.08E-1	9.13E-1
*MT2A*, Metallothionein 2 A	6.37E-1	9.13E-1
*left PG*	6.61E-1	9.13E-1
*left IOG*	6.96E-1	9.13E-1
*ADGRE1*, Adhesion G protein-coupled receptor E1	7.21E-1	9.13E-1
*PPT1*, Palmitoyl-protein thioesterase 1	7.36E-1	9.13E-1
*MTMR6*, Myotubularin related protein 6	7.48E-1	9.13E-1
*TCN1*, Transcobalamin 1	7.49E-1	9.13E-1
*SLC6A4*, Solute carrier family 6 member 4	7.69E-1	9.13E-1
*DR1*, Down-regulator of transcription 1	8.05E-1	9.13E-1
*CEBPD*, CCAAT/enhancer binding protein (C/EBP) delta	8.09E-1	9.13E-1
*HLA-C*, Major Histocompatibility Complex class I C	8.12E-1	9.13E-1
*IL2RB*, Interleukin 2 receptor beta	8.25E-1	9.13E-1
*FYN*, FYN Proto-oncogene Src family tyrosine kinase	8.60E-1	9.13E-1
*ADGRG1*, Adhesion G protein-coupled receptor G1	8.94E-1	9.13E-1
*NFKBIA*, Nuclear factor of kappa light polypeptide gene enhancer in B-cells inhibitor alpha	9.20E-1	9.13E-1
*S100A8*, S100 calcium binding protein A8	9.46E-1	9.13E-1

### Correlation of imaging and RNA data blocks

**Fig. 1:**
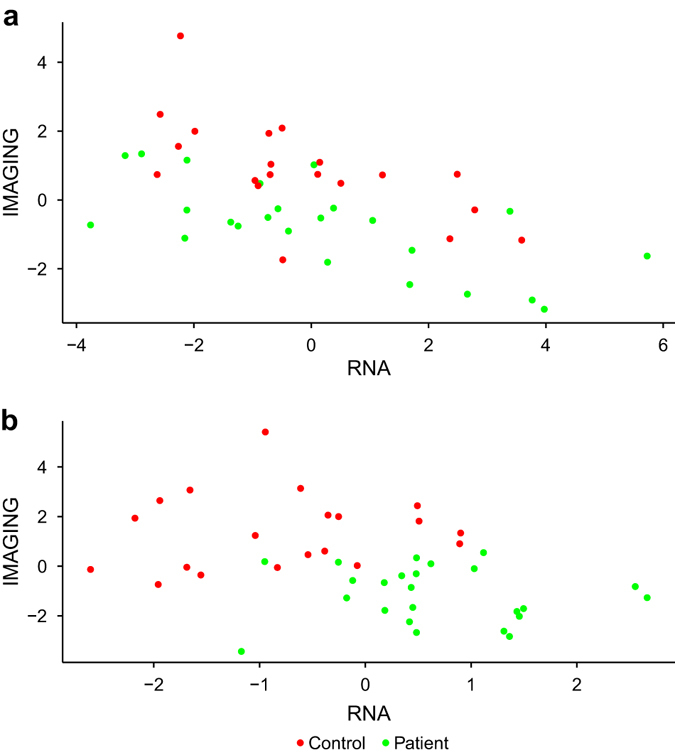
RGCCA plots in an unsupervised (**a** not including the factor patient vs. control) and in a supervised manner **b** to visualize how the blocks of gene expression (RNA) and imaging data (IMAGING) may structurally correlate to separate the SCZ patients (*green dots*) from the healthy controls (*red dots*)

To visualize how the blocks of gene expression and imaging data may structurally correlate to separate the SCZ patients from the healthy controls, we applied Regularized Generalized Canonical Correlation Analysis (RGCCA) first in an “unsupervised” manner. The analysis was thus restricted to the two blocks of imaging and RNA data and did not include the factor patient vs. control. Then, for comparison, we applied RGCCA in a “supervised” manner so that a third block, containing the binary variable describing the clinical status was incorporated (see Fig. 1a and b, respectively). Obviously, finding a classifier hyperplane representing separation efficiency would be much easier in the latter case, and of note, segregation of patients and controls is mostly due to a differential spreading of individuals on the vertical axis representing the imaging contribution. Therefore, as estimated by the above differential analysis, multivariate analysis also suggests imaging data contribute much more than RNA data to differentiate patients from controls.

### SGCCA

**Fig. 2:**
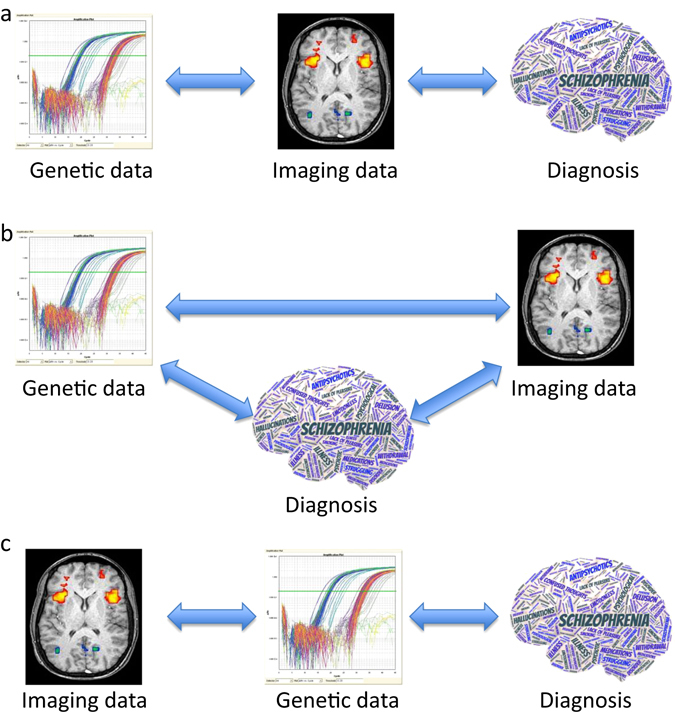
Three designs have been tested and named sequential **a**, complete **b** and reversed-sequential **c** to relate the RNA expression data, the imaging data and the clinical diagnosis

**Fig. 3:**
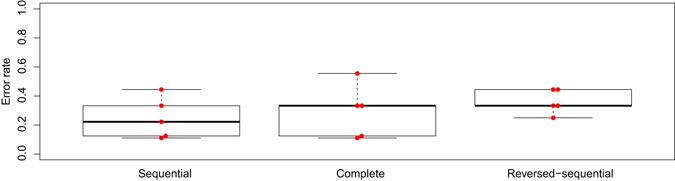
Boxplots of the error rates for the three tested designs. The *red dots* represent the actual error rates

The classical hypothesis of imaging genetics, which is depicted on Fig. 2a as a sequential model, states that imaging phenotypes are intermediate between genetics data and disease diagnosis. We proposed a first alternative hypothesis, named complete model, represented on Fig. 2b. The complete model would not impose any order in the contribution of genetic and imaging blocks of data to determine the subject phenotype (control vs. patient). We also tested the possibility of a reversed-sequential model (Fig. 2c), where transcriptional data would be intermediate between imaging data and the disease diagnosis. To test which model fits better with our biological data, we applied SGCCA to the three designs. The performance of the multiblock analysis was evaluated by an external cross validation loop. The classical test error rate computed by building a classification model on the latent components of the ROIs and the gene expression variables to predict the outcome phenotype (SCZ vs. control) with a Linear Discriminant Analysis. This test error rate is represented on Fig. 3, after removing the effects of age, gender, and smoking. As we obtained the most robust performance of SGCCA with the sequential design, we proceeded to identify the discrete RNA and imaging signatures only with that design, as reported on Table 2.

**Table 2:** Variables selected with SGCCA after the cross-validation procedure

RNA signature	Mean	f^a^	Values^b^	Imaging signature	Mean	f^a^	Values^b^
***S100A10***	**0.40**	**4**	**0.30**; 0.00; **0.39**; **1.00**; **0.29**	**right STG**	**0.57**	**5**	**0.48**; **0.38**; **0.48**; **1.00**; **0.53**
***MTMR6***	**0.35**	**4**	**0.28**; **0.99**; 0.15; 0.00; **0.33**	**left STG**	**0.42**	**4**	**0.47**; **0.34**; **0.69**; 0.00; **0.58**
***ADGRE1***	**0.20**	**3**	**0.34**; 0.00; 0.07; 0.00; **0.57**	**left FG**	**0.29**	**4**	**0.40**; **0.24**; **0.47**; 0.00; **0.33**
***CXCR3***	0.16	**3**	0.13; 0.00; 0.13; 0.00; **0.56**	**right DLPFC**	**0.24**	**4**	**0.31**; **0.40**; 0.12; 0.00; **0.35**
***DR1***	0.15	**3**	**0.21**; 0.00; **0.33**; 0.00; **0.22**	**left DLPFC**	**0.23**	**4**	**0.27**; **0.43**; **0.24**; 0.00; **0.20**
***FTO***	0.12	**3**	0.19; 0.00; **0.34**; 0.00; 0.07	**dorsal ACC**	0.17	**3**	0.17; **0.36**; 0.00; 0.00; **0.31**
***MT2A***	0.12	**3**	**0.23**; 0.00; 0.19; 0.00; 0.17	**rostral ACC**	0.13	**3**	**0.22**; **0.36**; 0.00; 0.00; 0.09
***CX3CR1***	0.11	**3**	**0.21**; 0.12; **0.24**; 0.00; 0.00	**right FG**	0.07	2	**0.27**; 0.09; 0.00; 0.00; 0.00
***IL1B***	0.11	**3**	**0.25**; 0.00; 0.16; 0.00; 0.15	right PG	0.07	2	0.18; 0.17; 0.00; 0.00; 0.00
***IL2RB***	0.09	**3**	0.12; 0.00; **0.25**; 0.00; 0.09	right amygdala	0.05	2	0.07; 0.16; 0.00; 0.00; 0.00
*SRGN*	0.07	**3**	0.12; 0.00; 0.10; 0.00; 0.11	left amygdala	0.04	2	0.11; 0.07; 0.00; 0.00; 0.00
***FYN***	0.12	2	0.10; 0.00; **0.50**; 0.00; 0.00	left PG	0.03	2	0.09; 0.07; 0.00; 0.00; 0.00
***TCF4***	0.08	2	**0.26**; 0.00; **0.20**; 0.00; 0.00	left IOG	0.02	2	0.08; 0.04; 0.00; 0.00; 0.00
***PPT1***	0.08	2	**0.35**; 0.00; 0.07; 0.00; 0.00	right IOG	0.02	2	0.06; 0.02; 0.00; 0.00; 0.00
***IFITM3***	0.08	2	**0.30**; 0.00; 0.12; 0.00; 0.00	right thalamus	0.01	2	0.02; 0.04; 0.00; 0.00; 0.00
***CEBPD***	0.08	2	0.17; 0.00; **0.21**; 0.00; 0.00	left thalamus	0.01	2	0.00; 0.03; 0.00; 0.00; 0.00
***MT1X***	0.06	2	0.08; 0.00; 0.00; 0.00; **0.23**				
*S100A8*	0.05	2	0.12; 0.00; 0.11; 0.00; 0.00				
*PRF1*	0.03	2	0.08; 0.00; 0.09; 0.00; 0.00				
*SLC6A4*	0.02	2	0.02; 0.00; 0.10; 0.00; 0.00				
*ELK1*	0.02	2	0.00; 0.00; 0.11; 0.00; 0.00				
*HLA-C*	0.02	2	0.08; 0.00; 0.02; 0.00; 0.00				
*ADGRG1*	0.02	2	0.10; 0.00; 0.00; 0.00; 0.00				
*EOMES*	0.02	2	0.09; 0.00; 0.01; 0.00; 0.00				
*MAPK6*	0.01	2	0.02; 0.00; 0.02; 0.00; 0.00				
*RAB6A*	0.03	1	0.15; 0.00; 0.00; 0.00; 0.00				
*TCN1*	0.02	1	0.12; 0.00; 0.00; 0.00; 0.00				
*G3BP2*	0.02	1	0.12; 0.00; 0.00; 0.00; 0.00				
*NFKBIA*	0.02	1	0.09; 0.00; 0.00; 0.00; 0.00				

^a^Number of occurrence of values >0 in the 5-fold cross validation; ^b^Only the absolute values were reported.

Genes and ROIs for which at least one value is ≥ 0.20 are indicated in bold. Shaded parts of the table highlight genes and ROIs with at least 3 out of 5 values >0.

From the 33 candidate gene signatures we entered in the model, 29 participated at least weakly to the outcome phenotype. More specifically, 11 genes (indicated in shaded columns on the left side of Table 2) conveyed more than in 60% of the cross-validation folds to the SCZ diagnosis, the more robust contributions being provided by *S100A10* and *MTMR6*. Regarding the imaging data, all tested ROI could contribute to the final outcome phenotype, at least weakly. There too, no more than half of them reached a significant level to determine the diagnosis decision. Of note, bilateral regions were very informative, and comprised the ACC, the DLPFC, the FG and the STG (shaded columns on the right side of Table 2).

## Discussion

In the cascade from gene to cell, neural system and finally behavior, SCZ studies on the genetic component have been largely focused on individual or combined allelic polymorphisms. In this paradigm, emerged the neuroimaging intermediate phenotype.^4^ The main limit of this model is that such genetic information remains static within an individual along his life and then cannot alone reflect the interaction with environment. Main-effect approaches assume direct connection between genes and disorders. By contrast, the integration of environment interaction to the cascade of causality underlying a psychiatric/cognitive syndrome expects no direct gene-to-behavior association. Although abnormalities in SCZ patients have been linked genetic variants modulating mRNA expression in brain,^7^ the need for investigations taking into account the contribution of environment is persistent. For that purpose, the study of blood as a surrogate tissue to measure the gene x environment interaction in relation to imaging measurement in the same individual, within a short time frame (less than 2 h), is a major progress for investigators who challenged the dogma that mental disorders can only be explained restricting molecular exploration to the central nervous system. Indeed, recent works contribute to weaken such consideration.^7, 9, 12^ In line with these approaches, we conducted, to our knowledge, the first study aiming at predicting the SCZ phenotype by linking candidate gene transcripts expression in blood with measures of brain circuits and function.

To this day, only a handful studies considered RNA transcripts as the genetic basis in relation to imaging data so as to predict a neuropsychiatric phenotype.^8, 10, 12, 52–57^ Theoretically, genome-wide exploration of transcriptomic variations could enable to seek for novel genetic loci related to brain structure and function. However, such a task requires immense computational power, especially if whole brain data is considered. Therefore, as a preliminary proof of concept study, we favored a candidate-driven approach, which would confront somewhat equilibrated blocks of candidate gene transcripts expression values with ROIs fMRI data.

In our study, none of the 31 candidate gene transcripts expressed in blood revealed a significantly different pattern between SCZ patients and healthy controls after correcting for age, sex and smoking covariates. This result is in agreement with a lack of direct relation between a peripheral transcriptional expression and a complex behavior. Thus, in line with the dominant paradigm of imaging genetics, the expression of some of our gene candidates could be linked to intermediate brain phenotype from specific region of interest to predict a health or pathological phenotype. Remarkably, the gene candidates that provided the most successful transcriptional pattern for imaging genetics were *S100A10* and *MTMR6*. Though there is not much literature on the later, it is involved in the regulation of the Ca2^+^-activated K^+^ channel KCa3.1, apoptosis and autophagy in mammalian cells.^58^ *MTMR6* also seems to play a critical role in setting a minimum threshold for a stimulus to activate a T cell.^59^ *S100A10* is involved in serotonergic neurotransmission and synaptic plasticity. Its dysregulated expression in limbic structures, but also in immune blood cell subsets, has been associated to pathological behavior or psychoactive drug response such as antidepressant.^60^

Among key fMRI regions, not surprisingly, the STG appears at the top of the list. Indeed, consistent evidence shows that the STG plays an important role in the pathophysiology of SCZ and this brain region connects to multiple structures of the limbic system, the thalamus and regions in the prefrontal cortex, all of which are also involved in SCZ. It has been proposed that abnormalities in this region could be related to a number of core symptoms in SCZ such as auditory hallucinations, emotional processing deficits or impaired social cognition.^61–63^ Previous findings support the hypothesis that STG changes in SCZ are not due to medication and represent a vulnerability marker of the illness.^64–66^ It is therefore coherent, in our model, to find activity in this region robustly linked to both gene expression and diagnosis.

The DLPFC is another key region highly involved in the physiopathology of SCZ.^67, 68^ This region is implicated in high-level cognitive skills such as executive functions, working memory or affect regulation.^51^ The VAAT task was thus expected to strongly tap this region through both cognitive control and emotion regulation processes. The prominent influence of the DLPFC in our model could be explained by the fact that the prefrontal cortex is the cerebral region with the most delayed ontogeny,^67^ thus more affected by environmental factors.^69^ Together, these findings point to an interaction between specific genes and environmental factors in SCZ, leading to DLPFC alterations.

The left FG is specifically involved in face recognition and abnormal activity in this region has been related to abnormal face recognition in SCZ.^70, 71^ Previous MRI studies in SCZ have demonstrated reduced gray matter volume in this region^72^ and suggest a progressive process, specific to this region.^73^ Finally, the ACC is a central region of the cortical-subcortical circuits highly involved in cognitive control, decision-making and affects regulation.^74^ Numerous studies have shown anatomic, functional and electrophysiological abnormalities in the ACC in SCZ^75^ and point to a specific and crucial involvement of this region in the psychopathology of this illness.^76, 77^

Though, several limitations to our present work must be cited. First, the sample size of our cohort was small. Ideally, a power analysis would be required to determine the optimal sample size. However, because the measurements we made are novel, we could neither estimate their variability nor their expected mean values, and thus, a sample size calculation is beyond our current knowledge. For such a reason, we cannot exclude a potential overestimation of the effects that have been detected, and there is a need for a replication in an independent sample. Second, while we focused on mRNA of candidate genes, recent works demonstrated a master role of noncoding RNAs such as microRNAs (miRNAs) in altering behavior at the basis of psychiatric disorders.^78^ As for example, miR-137, one of the very few genetic loci strongly associated to SCZ, exhibits a brain regional pattern of expression. Moreover, miR-137’s targets are significantly enriched for association with activation in the dorsolateral prefrontal cortex.^79^ Third, gene expression reflects in general the sum of multiple transcripts that are generated through the process of mRNA alternative splicing. One might consider focusing on specific isoform to better reflect gene x environment duality of information in the imaging genetics paradigm. In fact, it has been shown that dysfunctional gene splicing is a contributor to neuropsychiatric disorders.^80, 81^ Fourth, the gene expression could be influenced by genetic variants and it would be very informative that further investigations consider the combination of imaging data with mRNA levels of expression but also the profile of common single nucleotide polymorphisms as well as epigenetic marks (such as miRNA level of expression or DNA methylation profiles), especially in the context of the comparison of SCZ patient to their unaffected siblings in addition to unrelated healthy controls.

Finally, the choice of ROI in this emotional task could be considered as arbitrary. The regions were selected on the basis of their involvement in the two main aspects of the task, visual and emotion processing. These areas could have been broadened, in particular with other regions known to be involved in emotion processing (insula, orbitofrontal cortex). We restrained our choice to regions showing a strong main effect of task. Interestingly, the selected ROIs included both regions previously pointed in the physiopathology of SCZ and other regions never incriminated. It is interesting to see here that the regions selected by our model have been the most strongly related to SCZ illness (STG, DLPFC) although they have not emerged from our first classic statistical analysis. This comforts the applicability of the SGCCA analysis in this type of data. Finally, given the central role of emotional dysregulation in many, if not all, psychiatric illnesses, better characterization of the neural correlates underlying emotion processing and its link with gene expression could constitute a relevant dimensional approach in all psychiatric phenotypes.

## Methods

### Population

29 SCZ patients and 33 age- and sex-matched healthy volunteers completed the study. Before entering the study, subjects underwent a medical interview and examination. All patients met DSM-5 criteria for SCZ,^82^ were stabilized by antipsychotic monotherapy by Aripiprazole or Risperidone for at least 6 weeks and met remission criteria.^83^ They were either inpatients hospitalized in a general public mental hospital or outpatients regularly followed by a psychiatrist. Healthy controls (HCs) were recruited through advertising in the local community of Marseille. They were matched to patients on gender, age and education. The non-patient version of the Structured Clinical Interview for DSM-V (SCID) was used to ensure the absence of psychiatric disorder or psychiatric history in the HC participants. In SCZ patients and HC groups, exclusion criteria were the following: MRI contraindications; history of head injury or neurological disorder, concomitant major somatic comobidity, or drug abuse. All participants had to be right-handed according to the Edinburgh Handedness Inventory^84^ and had normal or corrected-to normal vision.

Data from nine participants (3 patients and 6 controls) were removed because of excessive head motion, anomalies detected on anatomical scans, or visible artifacts in functional images. Thus, the final analyses included data from 26 patients and 26 healthy controls (Supplementary Table 3).

This study was conducted in accordance with the principles of the declaration of Helsinki. Approval was obtained from the local ethics committee (Comité de protection des personnes Sud Méditerranée I, Marseille, registered under ref. 09,61 MS 2) and each participant gave informed written consent before entering the study.

### Experimental paradigm

The experimental task (Variable Attention and congruency Task [VAAT] was previously described^51^). Briefly, participants were presented with images composed of two parts. The central part of the image displayed photographs of faces expressing positive (joy) or negative (fear, disgust, or anger) emotion, extracted from the NimStim Face stimulus set.^85^ The peripheral surround, upon which face images were superimposed, represented scenes with pleasant or unpleasant emotional content, extracted from IAPS files.^86^ Subjects were asked to focus on the part of the image framed in green (either the central face or the peripheral scene) and determine its emotional content (pleasant vs. unpleasant) by pressing the corresponding key.

The task consisted of 3 × 2 conditions varying according to emotional valence (positive or negative), emotional congruency (same or different emotional content in the face and the scene), and attentional load (attention focused on the face [low attention] or on the scene [high attention]). The VAAT had a mixed event-related/block design. The blocks began by an instruction panel (displayed during 1400 ms) specifying upon which part of the image the subject had to focus during the block, followed by 4 experimental trials, each lasting 3000 ms, during which time subjects provided their response. The valence parameter varied from trial to trial whereas the congruency and attention parameters varied from block to block. The inter-stimulus interval (ISI) and inter-block interval (IBI) were randomly jittered ranging from 1 to 1.8 s for the ISI and from 1.2 to 2 s for the IBI, with a respective mean of 1.4 and 1.6 s. Block order was randomized within sessions, and the order of the sessions was counterbalanced across subjects.

### MRI acquisition

Data were acquired on a 3-T MEDSPEC 30/80 AVANCE imager (Bruker). After an initial localizing scan, functional data were acquired using a T2*-weighted gradient-echoplanar imaging sequence (number of repetition = 200; TR = 2400 ms; TE = 30 ms; FOV = 19.2 × 19.2; 64 × 64 matrix; flip angle 81.6°; voxel size 3 × 3 × 3 mm³; slices = 36) along the anterior–posterior commissure plane with a continuous slice thickness of 3 mm. Following the functional magnetic resonance imaging (fMRI) scans, high-resolution anatomical images were acquired with a sagittal T1-weighted MP-RAGE sequence (TR = 9.4 ms; TE = 4.42 ms; TI = 800 ms; 256 × 256 × 180 matrix; flip angle 30°, voxel size 1 × 1 × 1 mm³).

### fMRI data analysis

Data analysis was conducted as previously described.^51^ Prior to analysis, the quality of the functional images was assessed using tsdiffana (http://imaging.mrc-cbu.cam.ac.uk/imaging/DataDiagnostics). Functional images were subjected to spike artifact detection. The quantitative quality indicators (signal-to-noise ratio, scaled variance, scaled mean voxel, slice by slice variance) were examined to ensure the stability of the signal over time and the lack of abrupt variation between successive slices. All data were analyzed using SPM8 software (Wellcome department of Cognitive Neurobiology, University College London; http://www.fil.ion.ucl.ac.uk/spm/software/spm8). The first 4 volumes of each session, corresponding to signal stabilization, were excluded from the analysis. The remaining scans were corrected for differences in slice acquisition time. To reduce the effect of head motion, whole images were realigned to the mean scan of each session. Realignment plots were examined to ensure the absence of excessive movements during the scan. Data were discarded from further analysis if movements in any axis were superior to 3 mm and/or 2°. The structural scan was co-registered to the functional images, and all images were transformed into a standardized coordinate system corresponding to the Montreal Neurological Institute (MNI) space. The normalized images were spatially smoothed with an isotropic Gaussian kernel (full width at half maximum of 6 mm). Finally, each preprocessing step was check using the Check Registration function implemented in SPM.

The preprocessed functional images were analyzed using an event-related approach. Hemodynamic responses were modeled using a canonical function and convolved with the onsets and durations of each condition to form the general linear model. Six movement parameters were included in the analysis as regressors of no interest. A 128 s high-pass filter was applied to the data to remove low-frequency noise. For each participant, first-level contrast images were calculated to estimate BOLD signal changes due to variation in emotional valence (negative vs. positive valence conditions), emotional congruency (incongruent vs. congruent conditions), and attentional level (attention to the scene [high] vs. attention to the face [low]). The first-level contrast images were then entered into a second-level one-sample t-test with a random effects statistical model to examine the main effects of the task at the group levels.

We used a region of interest (ROI) approach, obtained from the 3 conditions of the fMRI paradigm, focusing on regions involved in the two main aspects of the task, visual processing: right and left thalamus (Thalamus_R/L), right and left inferior occipital gyrus (Occipital_Inf_R/L), right and left fusiform gyrus (Fusiform_R/L), right and left para hippocampal gyrus (Parahippocampal_R/L), and right and left superior temporal gyrus (Temporal_Sup_R/L), as well as areas previously implicated in emotion processing: the right and left amygdala (Amygdala_R/L), dorsal and rostral ACC (Cingulum_Ant_R/L), and right and left DLPFC (Frontal_Inf_Tri_R/L + Frontal_Mid_R/L). These 16 ROIs were anatomically defined using the Automated Anatomical Labeling software implemented in the WFU PickAtlas.^87^ We identified healthy group local maxima coordinates within each of these ROIs at a statistical threshold of *P* < 0.001 voxel-wise (uncorrected for multiple comparisons). Using the MARSBAR toolbox,^88^ we built 10-mm radius spheres (5 mm for the amygdala) centered around these coordinates. We then extracted for each subjects the mean activity beta value within each sphere that we entered in the RGCCA model.

### Blood mRNA extraction

8–10 ml of venous blood was collected from fasting SCZ patients and matched-HCs in EDTA tubes between 7:00 and 9:00 a.m., I.E. just preceding MRI testing, and processed within 2 h. Peripheral blood mononuclear cells (PBMCs) were isolated from the blood by Ficoll density centrifugation. Total RNA was extracted from the PBMCs with the mirVana miRNA isolation kit (Ambion, Austin, TX) according to the manufacturer’s protocol. RNA concentration was determined using a nanodrop ND-1000 spectrophotometer (NanoDrop Technologies, Wilmington, DE). RNA integrity was assessed on an Agilent 2100 Bioanalyzer (Agilent Technologies, Santa Clara, CA), and only samples that exhibited an RNA integrity number (RIN) superior to 8 were further processed. 7 samples were excluded (1 patient and 6 controls) because of RIN <8. For the genetic imaging study, the samples corresponding to participants whose data could not be analyzed for fMRI were also excluded for gene expression. Finally, gene expression was evaluated on 25 SCZ patients and 20 healthy controls.

### Candidate gene selection

To select candidate genes, we focused on genes reported in the literature to underlie SCZ pathogenesis and emotion regulation. According to our previous genome-wide microarray data obtained on HCs and major depressive patients,^89^ some candidate genes were excluded because of a too low level of expression in PBMCs. Based on a meta-analysis we previously conducted on SCZ and HC samples,^19^ we retained 11 genes dysregulated both in blood and brain (*ADGRG1, CX3CR1*, *DR1*, *FYN*, *G3BP2*, *HLA-A*, *MAPK6*, *MTMR6*, *RAB6A*, *S100A8*, *UBE2D2*), 8 genes dysregulated in blood (*CXCR3*, *EOMES*, *FTO*, *GYG1*, *IL2RB*, *PRF1*, *TCF4*, *TCN1*), and 14 genes dysregulated in brain (*ABL1*, *ADGRE1*, *CEBPD*, *ELK1*, *IFITM3*, *HLA-C*, *IL1B*, *MT1X*, *MT2A*, *NFKBIA*, *PPT1*, *S100A10*, *SLC6A4*, *SRGN*) (Supplementary Table 4). In total, we retained 38 genes to assay transcriptional expression (Supplementary Table 5).

### Real-time RT-PCR for candidate gene expression

1.6 μg of RNA was reverse transcribed with the High Capacity cDNA archive kit (Applied Biosystems, Foster City, CA, USA). For a first set of 32 genes, 200 ng of the resulting cDNA were combined with a TaqMan® universal PCR Master Mix (Applied Biosystems) and 32 PCR reactions were simultaneously performed in triplicate on an ABI PRISM 7900HT thermocycler using tLDA technology according to manufacturer recommendations (Applied Biosystems). For a second set of 11 genes, individual reaction were performed in triplicate with 50 ng of cDNA combined with TaqMan® universal PCR Master Mix. For each candidate gene tested, primer sets and probes were selected using the web portal of the manufacturer (Applied Biosystems, see Supplementary Table 5). In addition, we used 5 genes as references for the level of expression: (i), *GAPDH* (very highly expressed gene, imposed by the manufacturer), (ii) *G3BP2* (highly expressed), (iii) *DDX47*, *CRYL1 (*moderately expressed), and (iv) *SV2A* (weakly expressed). After verifying on DataAssist software (Applied Biosystems, v3.0) that our 5 selected reference genes were stably expressed among all the samples from MDE patients and controls tested by RT-qPCR, we set-up 5 windows of expression intensities to normalize target gene expression for tLDA data (Ct < 22; 22 < Ct < 24; 24 < Ct < 25.5; 25.5 < Ct < 28.5; Ct > 30) (Supplementary Table 5). *CRYL1* was universally used as a reference gene for the individual amplification of the second set of genes. The expression level of each candidate gene was calculated as 2^-ΔΔCt^ with DataAssist. In this method, each candidate gene is quantified relative to the expression of one or two reference genes, to calculate a proximal level (i.e. difference between the target and the reference mRNA <2 Ct) of expression compared to the target gene. We compared each amplification to a calibrator sample (the mean of the samples from the control subjects).

### MANCOVA

MANCOVA^90^ is a multivariate extension of ANOVA that allows one to assess the impact of a qualitative variable on several response variables while taking into account the variations of several covariates. In our case, the response variables are either the transcripts quantifications or the imaging variables and the covariates are age, gender and smoking status.

### SGCCA

By contrast to the above methods that have been used extensively by other investigators, the following analyses are original in the context of imaging genetics and rely on a general framework for multiblock component methods called Regularized Generalized Canonical Correlation Analysis (RGCCA), that was previously published^91, 92^ and assessed on real data.^93–95^ RGCCA is a multivariate method adapted to the analysis of several blocks of variables. In a nutshell, RGCCA aims to extract the linear relationships that best explain the correlated structure across datasets. In this formalism, each dataset is called a block and represents a set of measurements obtained on the same individuals: in our case, we have three different blocks, one phenotypic block (e.g. age, gender, smoking status, clinical status etc.), one block of gene expression data and one block of imaging data. Our method reduces the RNA and imaging blocks of variables to a few meaningful components, akin to principal components, that are computed such that they capture the variability of their own block while taking into account the variability in the other blocks. Being a component-based approach, RGCCA requires the estimation of block components ${{\bf{y}}_{\bf{j}}} = {{\bf{X}}_j}{{\bf{a}}_j},j = 1, \ldots ,J$ obtained such that (i) block components explain well their own block and/or (ii) block components that are assumed to be connected are highly correlated. The optimization process behind RGCCA is fully described in the following equation:

$$\begin{array}{l}\\ \mathop {{\max }}\limits_{{{\bf{a}}_{\rm{.}}}} \mathop {\sum }\limits_{j,k} {C_{j,k}}g\left( {{\rm{cov}}\left( {{{\bf{X}}_j}{{\bf{a}}_j},{{\bf{X}}_k}{{\bf{a}}_k}} \right)} \right)\\ \\ {\rm{such}}\,{\rm{that}}\,{\bf{a}}_j^ \top {{\bf{M}}_j}{{\bf{a}}_j} \le 1,\forall j,\\ \end{array}$$

where **X**_*j*_ denotes one of the data blocks, **a**_*j*_ is the vector of the linear weights applied to block *j* to compute the *j* th block component, *g* is a so-called scheme function, usually the square or the absolute value function, **M** *j* is a square matrix realizing a compromise between constraining the norm of the weights or constraining the variance of the block components. *C* represents the binary matrix of connections between the blocks. Indeed, through the so-called design matrix *C*, RGCCA can process a priori information defining which blocks are supposed to be linked to one another, thus reflecting hypotheses about the biology underlying the data blocks. The term “generalized” in the acronym of RGCCA embraces at least three notions. The first one relates to the generalization of two-block methods – including Canonical Correlation Analysis,^96^ Interbattery Factor Analysis^97^ and Redundancy Analysis^98^ to three or more sets of variables. The second one relates to the ability of taking into account some hypotheses on between block connections: the user decides which blocks are connected and which are not. The third one relies on an optimal compromise between correlation and covariance-based criteria. In this work, we were interested by biomarker discovery, we therefore use SGCCA, (sparse RGCCA),^25^ a variation of RGCCA that allows the identification of the most relevant features within each block. As component-based methods, RGCCA and SGCCA can provide the user with graphical representations to visualize the sources of variability within blocks and the amount of correlation between blocks. Finally, unlike MANCOVA, RGCCA and SGCCA are able to cope with a high number of variables.

Before applying SGCCA, all the variables were standardized and adjusted by residualization (before preprocessing) for the commonly examined confounding factors age and gender but also for the smoker status. Rates of smokers in SCZ patients are multiple times the rates for regular smocking in the general population, as well as those with other disorders^99^ and a blood transcriptional signature of the smoking status has been demonstrated. Such signature is reported to be different in male and female and affects some of the candidate genes we selected.^100–102^ This procedure is based on a test error rate measured on the test sets with a Linear Discriminant Analysis, similarly to what was previously done in the original article.^25^ Finally, we also undertook a 5-fold cross validation procedure where each fold yields a different sets of leadings, allowing us to assess their variability and especially the number of times a candidate biomarker was selected (meaning that its corresponding loading was different from zero). We considered that a candidate biomarker was robust if it was selected more than 3 times out of 5. The sparsity parameters controlling the number of selected variables were set using a cross validation procedure.

RGCCA and SGCCA are implemented in an R package freely available on the Comprehensive R Archive Network’s website (https://cran.r-project.org/package=RGCCA).

### Data availability

All imaging and genetic data used for multiblock component methods are available upon request.

## Electronic supplementary material

Supplementary Table 1

Supplementary Table 2

Supplementary Table 3

Supplementary Table 4

Supplementary Table 5
