# Supplementary material for: Modeling a linkage between blood transcriptional expression and activity in brain regions to infer the phenotype of schizophrenia patients
Source: NPJ Schizophr. 2017 Sep 7;3:25. doi: 10.1038/s41537-017-0027-3 (PMC5589880; doi:10.1038/s41537-017-0027-3)
Supplement: Supplementary file 1 — Supplementary Table 1 [file 41537_2017_27_MOESM1_ESM.docx]

**Supplementary Table 1:** MANCOVA on the block of functional imaging data.

| Factor | DF | Pillai | approx F | num Df | den Df | Pr(>F) |
| --- | --- | --- | --- | --- | --- | --- |
| Group | 1 | 0.6383 | 2.647 | 16 | 24 | 0.01521 |
| Age | 1 | 0.3043 | 0.6560 | 16 | 24 | 0.8067 |
| Gender | 1 | 0.4993 | 1.496 | 16 | 24 | 0.1813 |
| Smoking | 1 | 0.3834 | 0.9327 | 16 | 24 | 0.5476 |
| Residuals | 39 | NA | NA | NA | NA | NA |
