# Supplementary material for: Modeling a linkage between blood transcriptional expression and activity in brain regions to infer the phenotype of schizophrenia patients
Source: NPJ Schizophr. 2017 Sep 7;3:25. doi: 10.1038/s41537-017-0027-3 (PMC5589880; doi:10.1038/s41537-017-0027-3)
Supplement: Supplementary file 2 — Supplementary Table 2 [file 41537_2017_27_MOESM2_ESM.docx]

**Supplementary Table 2:** MANCOVA on the block of candidate gene transcriptional data.

| Factor | DF | Pillai | approx F | num Df | den Df | Pr(>F) |
| --- | --- | --- | --- | --- | --- | --- |
| Group | 1 | 0.9412 | 1.779 | 36 | 4 | 0.3084 |
| Age | 1 | 0.8480 | 0.6197 | 36 | 4 | 0.8081 |
| Gender | 1 | 0.9335 | 1.56 | 36 | 4 | 0.3632 |
| Smoking | 1 | 0.9375 | 1.666 | 36 | 4 | 0.335 |
| Residuals | 39 | NA | NA | NA | NA | NA |
