# Supplementary material for: Modeling a linkage between blood transcriptional expression and activity in brain regions to infer the phenotype of schizophrenia patients
Source: NPJ Schizophr. 2017 Sep 7;3:25. doi: 10.1038/s41537-017-0027-3 (PMC5589880; doi:10.1038/s41537-017-0027-3)
Supplement: Supplementary file 3 — Supplementary Table 3 [file 41537_2017_27_MOESM3_ESM.docx]

**Supplementary Table 3:** Cohorts characteristics.

| Group | Control | SCZ | p-value |
| --- | --- | --- | --- |
| Age (mean years ± SD) | 33.0 ± 7.9 | 32.3 ± 8.8 | 0.767^a^ |
| Gender (F/M) | 9/17 | 9/17 | 1.000^b^ |
| Smoking (Yes/No) | 14 /12 | 18/8 | 0.089^b^ |
| Aripiprazole/Risperdone |  | 15/11 |  |

^a^ Student’s t-test.

^b^ χ^2^ test.
