# Supplementary material for: Modeling a linkage between blood transcriptional expression and activity in brain regions to infer the phenotype of schizophrenia patients
Source: NPJ Schizophr. 2017 Sep 7;3:25. doi: 10.1038/s41537-017-0027-3 (PMC5589880; doi:10.1038/s41537-017-0027-3)
Supplement: Supplementary file 4 — Supplementary Table 4 [file 41537_2017_27_MOESM4_ESM.docx]

**Supplementary Table 4: P-value of candidate genes in SCZ meta-analysis**

| Symbol | Name | P_Blood_ | P_Post Mortem_ |
| --- | --- | --- | --- |
| **Genes dysregulated in blood and brain** | | | |
| *ADGRG1* | Adhesion G protein-coupled receptor G1 | 1.46E-07 | 6.27E-07 |
| *FYN* | FYN Proto-oncogene Src family tyrosine kinase | 4.08E-06 | 4.53E-04 |
| *CX3CR1* | Chemokine (C-X3-C motif) receptor 1 | 1.21E-05 | 4.18E-07 |
| *S100A8* | S100 calcium binding protein A8 | 1.42E-04 | <2.10E-07 |
| *UBE2D2* | Ubiquitin-conjugating enzyme E2 D2 | 1.95E-04 | 1.05E-06 |
| *MTMR6* | Myotubularin related protein 6 | 1.59E-03 | 1.61E-04 |
| *MAPK6* | Mitogen-activated protein kinase 6 | 1.69E-03 | 1.37E-02 |
| *HLA-A* | Major Histocompatibility Complex Class I A | 2.20E-03 | 5.83E-04 |
| *DR1* | Down-regulator of transcription 1 | 2.36E-03 | <2.10E-07 |
| *G3BP2* | GTPase activating protein (SH3 domain) binding protein 2 | 4.27E-03 | 1.34E-05 |
| *RAB6A* | RAB6A, Member RAS oncogene family | 3.22E-02 | 7.07E-05 |
| **Genes dysregulated in blood only** | | | |
| *IL2RB* | Interleukin 2 receptor, beta | <1.46E-07 | 0.267 |
| *TCN1* | Transcobalamin 1 | <1.46E-07 | 0.411 |
| *PRF1* | Perforin 1 | <1.46E-07 | 0.492 |
| *EOMES* | Eomesodermin | <1.46E-07 | NA |
| *CXCR3* | Chemokine (C-X-C motif) receptor 3 | 7.43E-06 | 0.0789 |
| *TCF4* | Transcription factor 4 | 1.50E-04 | 0.589 |
| *FTO* | Fat mass and obesity associated | 6.46E-04 | 0.721 |
| *GYG1* | Glycogenin 1 | 1.91E-02 | 0.583 |
| **Genes dysregulated in brain only** | | | |
| *MT2A* | Metallothionein 2A | 0.0678 | <2.10E-07 |
| *CEBPD* | CCAAT/enhancer binding protein (C/EBP) delta | 0.121 | <2.10E-07 |
| *MT1X* | Metallothionein 1X | 0.457 | <2.10E-07 |
| *IFITM3* | Interferon induced transmembrane protein 3 | 0.992 | <2.10E-07 |
| *NFKBIA* | Nuclear factor of kappa light polypeptide gene enhancer in B-cells inhibitor alpha | NA | <2.10E-07 |
| *SRGN* | Serglycin | 0.302 | 2.17E-05 |
| *ELK1* | ELK1, member of ETS oncogene family | 0.0708 | 3.03E-05 |
| *S100A10* | S100 calcium binding protein A10 | 0.136 | 1.22E-04 |
| *ABL1* | c-abl oncogene 1, non-receptor tyrosine kinase | 0.485 | 6.79E-03 |
| *ADGRE1* | Adhesion G protein-coupled receptor E1 | 0.429 | 1.18E-02 |
| *PPT1* | Palmitoyl-protein thioesterase 1 | 0.448 | 1.35E-02 |
| *HLA-C* | Major Histocompatibility Complex class I C | 0.916 | 1.39E-02 |
| *IL1B* | Interleukin 1 beta | 0.117 | 1.83E-02 |
| *SLC6A4* | Solute carrier family 6 member 4 | 0.744 | 4.96E-02 |
| **Reference genes for RT-qPCR** | | | |
| *GAPDH* | Glyceraldehyde-3-phosphate dehydrogenase | 0.438 | 0.502 |
| *MBD4* | Methyl-CpG binding domain protein 4 | 0.221 | 1.36E-02 |
| *DDX47* | DEAD (Asp-Glu-Ala-Asp) box polypeptide 47 | 0.136 | NA |
| *CRYL1* | Crystallin lamda 1 | 0.439 | NA |
| *SV2A* | Synaptic vesicle glycoprotein 2A | 0.315 | 0.453 |
