# Supplementary material for: Modeling a linkage between blood transcriptional expression and activity in brain regions to infer the phenotype of schizophrenia patients
Source: NPJ Schizophr. 2017 Sep 7;3:25. doi: 10.1038/s41537-017-0027-3 (PMC5589880; doi:10.1038/s41537-017-0027-3)
Supplement: Supplementary file 5 — Supplementary Table 5 [file 41537_2017_27_MOESM5_ESM.docx]

**Supplementary Table 5: Candidate genes tested by RT-qPCR**

| Reference gene | Detector | Ref | Ct (Mean ± SD) |
| --- | --- | --- | --- |
|  | *HLA-C* | Hs00762610_s1 | 19.28 ± 0.398 |
| *GAPDH* | *HLA-A* | Hs01058806_g1 | 19.43 ± 0.351 |
|  | *S100A8* | Hs00374264_g1 | 20.58 ± 0.539 |
|  | *GAPDH* | Hs99999905_m1 | 21.07 ± 0.481 |
|  | *SRGN* | Hs01004159_m1 | 21.58 ± 0.486 |
| *GAPDH* | *NFKBIA* | Hs00153283_m1 | 22.62 ± 0.474 |
| *MBD4* | *PPT1* | Hs00165579_m1 | 23.32 ± 0.554 |
|  | *CX3CR1* | Hs00365842_m1 | 23.92 ± 0.655 |
|  | *MBD4* | Hs00187498_m1 | 24.51 ± 0.406 |
|  | *IFITM3* | Hs03057129_s1 | 24.53 ± 1.067 |
|  | *CEBPD* | Hs00270931_s1 | 24.86 ± 0.556 |
| *MBD4* | *G3BP2* | *Hs00907696_m1* | 25.00 ± 0.432 |
|  | *UBE2D2* | Hs00366152_m1 | 25.23 ± 0.464 |
|  | *MT2A* | Hs02379661_g1 | 25.41 ± 0.732 |
|  | *DR1* | Hs00172424_m1 | 26.05 ± 0.414 |
|  | *DDX47* | Hs00275327_m1 | 26.46 ± 0.362 |
|  | *TCF4* | Hs00162613_m1 | 26.99 ± 0.437 |
|  | *MT1X* | Hs00745167_sH | 27.26 ± 0.509 |
| *DDX47* | *MTMR6* | Hs01103126_m1 | 27.28 ± 0.514 |
| *CRYL1* | *CRYL1* | Hs00211084_m1 | 27.49 ± 0.392 |
|  | *ADGRE1* | Hs00173562_m1 | 27.61 ± 0.506 |
|  | *RAB6A* | Hs00818388_m1 | 27.86 ± 0.488 |
|  | *IL1B* | Hs00174097_m1 | 28.47 ± 1.199 |
| *SV2A* | *SLC6A4* | Hs00169010_m1 | 31.10 ± 0.810 |
|  | *SV2A* | Hs00372069_m1 | 31.79 ± 0.624 |
|  | *ABI1* | Hs00178550_m1 | *Ct>35* |
|  | *GYG1* | *Hs00366308_m1* | *Ct>35* |
|  | *S100A10* | Hs00237010_m1 | 22.66 ± 0.530 |
|  | *ELK1* | Hs00901847_m1 | 27.01 ± 0.345 |
| *CRYL1* | *CRYL1* | Hs00211084_m1 | 27.04 ± 0.371 |
|  | *MAPK6* | Hs00833126_g1 | 28.71 ± 0.452 |
|  | *CXCR3* | Hs01847760_s1 | 28.96 ± 0.525 |
|  | *PRF1* | Hs00169473_m1 | 22.11 ± 0.593 |
|  | *FYN* | Hs00941600_m1 | 22.87 ± 0.374 |
| *CRYL1* | *IL2RB* | Hs01081697_m1 | 23.30 ± 0.554 |
|  | *ADGRG1* | Hs00173754_m1 | 23.77 ± 0.772 |
|  | *CRYL1* | Hs00211084_m1 | 25.72 ± 0.416 |
|  | *EOMES* | Hs00172872_m1 | 26.10 ± 0.621 |
|  | *FTO* | Hs01057145_m1 | 26.26 ± 0.413 |
|  | *TCN1* | Hs01055542_m1 | 30.12 ± 1.316 |
